# Supplementary material for: Postpartum Weight Change in Relation to Pre-Pregnancy Weight and Gestational Weight Gain in Women in Low-Income Setting: Data from the KITE Cohort in the Northern Part of Ethiopia
Source: Nutrients. 2021 Dec 28;14(1):131. doi: 10.3390/nu14010131 (PMC8746538; doi:10.3390/nu14010131)
Supplement: Supplementary file 1 [file nutrients-14-00131-s001.zip › nutrients-1513878-supplementary.pdf]

Supplementary Table S1. Summary of the nutritional characteristics of the women who successfully completed the study and included in the final analyses.

**Nutritional characteristics**	**Postpartum weight change in kg**					
	**Total n=585**	**Lost ≥1.0 n=106**	**Lost or gained <1.0 n=296**	**Gained 1.0–1.9 n=87**	**Gained 2.0–2.9 n=66**	**Gained ≥3.0 n=30**
Maternal height in cm, mean	157.3 (0.1)	158.9 (0.1)	1567.6 (0.1)	158.2 (0.1)	156.8 (0.1)	156.2 (0.1)
Pre-pregnancy weight in kg, mean	49.0 (6.9)	49.5 (6.4)	47.3 (6.4)	51.6 (6.8)	51.9 (7.6)	49.6 (7.5)
Weight at inclusion in kg, mean	51.4 (7.1)	51.6 (6.4)	49.6 (6.5)	54.6 (6.9)	54.9 (7.7)	52.8 (7.3)
Pre-pregnancy BMI in kg/m^2, mean	19.7 (2.0)	19.6 (1.8)	19.2 (1.8)	20.6 (2.1)	21.1 (2.0)	20.2 (2.1)
BMI at inclusion in kg/m^2, mean	20.7 (2.1)	20.4 (1.8)	20.1 (1.9)	21.8 (2.1)	22.3 (2.1)	21.6 (2.1)
MUAC at inclusion in cm, mean	22.6 (2.0)	22.5 (1.7)	22.1 (1.8)	23.4 (2.1)	23.8 (2.0)	23.1 (2.1)
Hemoglobin in g/dL, mean (n=550)	12.0 (1.6)	11.3 (1.4)	11.6 (1.6)	12.8 (1.4)	13.1 (1.3)	13.0 (1.5)
MUAC at 32 to 36 wks in cm, mean	22.8 (2.0)	22.7 (1.8)	22.3 (1.8)	23.6 (2.1)	24.0 (2.0)	23.3 (2.1)
Weight at 32 to 36 wks in kg, mean	59.7 (7.9)	58.1 (6.8)	57.3 (7.1)	64.2 (6.9)	65.3 (8.0)	64.2 (7.6)
Gestational weight gain in kg, mean	10.8 (2.3)	8.6 (1.5)	10.1 (1.6)	12.6 (1.2)	13.4 (1.2)	14.6 (0.8)
Postpartum MUAC in cm, mean	23.1 (2.5)	22.0 (2.0)	22.3 (2.1)	24.6 (2.4)	25.6 (2.3)	25.2 (2.4)
Postpartum weight in kg, mean	49.4 (7.2)	47.8 (6.3)	47.3 (6.4)	53.1 (6.8)	54.3 (7.6)	53.2 (7.4)
Postpartum BMI in kg/m^2, mean	19.9 (2.2)	18.9 (1.8)	19.2 (1.8)	21.2 (2.1)	22.0 (2.0)	21.8 (2.1)
Postpartum weight change in kg, mean	0.42 (1.5)	-1.7 (0.6)	0.08 (0.5)	1.5 (0.3)	2.4 (0.3)	3.7 (0.6)
Pre-pregnancy BMI, n (%)						
Underweight	213 (36.4)	37 (17.4)	143 (67.1)	18 (8.5)	8 (3.8)	7 (3.3)
Normal weight	372 (63.6)	69 (18.6)	153 (41.1)	69 (18.6)	58 (15.6)	23 (6.2)
Gestational weight gain, n (%)						
Inadequate weight gain	364 (62.2)	103 (28.3)	240 (65.9)	15 (4.1)	5 (1.4)	1 (0.3)
Adequate weight gain	221 (37.8)	3 (1.4)	56 (25.3)	72 (32.6)	61 (27.6)	29 (13.1)
Postpartum BMI, n (%)						
Underweight	194 (33.2)	55 (28.4)	119 (61.3)	14 (7.2)	4 (2.1)	2 (1.0)
Normal weight	391 (66.8)	51 (13.0)	177 (45.3)	73 (18.7)	62 (15.9)	28 (7.2)
